# A Comprehensive Case Study of Data-Driven Methods for Robust Aircraft Sensor Fault Isolation

**DOI:** 10.3390/s21051645

**Published:** 2021-02-26

**Authors:** Nicholas Cartocci, Marcello R. Napolitano, Gabriele Costante, Mario L. Fravolini

**Affiliations:** 1 Department of Engineering, University of Perugia, Via G. Duranti, 67, 06125 Perugia, Italy; nicholas.cartocci@unipg.it (N.C.); gabriele.costante@unipg.it (G.C.); 2 Department of Mechanical and Aerospace Engineering, West Virginia University, Morgantown, WV 26506-6106, USA; marcello.napolitano@mail.wvu.edu

**Keywords:** data-driven fault diagnosis, robust residual generation, fault isolation and estimation, Bayesian filtering, aircraft safety, flight data

## Abstract

Recent catastrophic events in aviation have shown that current fault diagnosis schemes may not be enough to ensure a reliable and prompt sensor fault diagnosis. This paper describes a comparative analysis of consolidated data-driven sensor Fault Isolation (FI) and Fault Estimation (FE) techniques using flight data. Linear regression models, identified from data, are derived to build primary and transformed residuals. These residuals are then implemented to develop fault isolation schemes for 14 sensors of a semi-autonomous aircraft. Specifically, directional Mahalanobis distance-based and fault reconstruction-based techniques are compared in terms of their FI and FE performance. Then, a bank of Bayesian filters is proposed to compute, in flight, the fault belief for each sensor. Both the training and the validation of the schemes are performed using data from multiple flights. Artificial faults are injected into the fault-free sensor measurements to reproduce the occurrence of failures. A detailed evaluation of the techniques in terms of FI and FE performance is presented for failures on the air-data sensors, with special emphasis on the True Air Speed (TAS), Angle of Attack (AoA), and Angle of Sideslip (AoS) sensors.

## 1. Introduction

Fault Diagnosis (FD) is a term that describes the general problem of detecting and isolating faults in physical components or in the instrumentation of a system [1]. A broad class of FD methods is based on the concept of Analytical Redundancy (AR) [2]. The essence of AR methods lies in the comparison of the behavior of the system with the response of a mathematical model. Inconsistency between the actual and model response is considered a symptom of a possible failure. An FD scheme elaborates this information in real time to detect and isolate faults.

Mathematical models play a central role in AR-based FD; these are derived from either physical laws governing the dynamics of the system or are inferred directly from experimental data exploiting system identification techniques [3].

In the last 30 years, FD has been widely investigated, and many techniques have been proposed. Considering model-based approaches, the main research directions can be coarsely categorized as fault detection filters, parity-space schemes, diagnostic observers, and parameter estimation [1]. Classic methods dealing with model-based approaches can be found in [4,5,6,7,8,9]. More recently, approaches based on optimal criteria [10,11] and extensions to nonlinear [12], hybrid [13], and distributed systems [14] have been proposed. A collection of successful applications of model-based FD was presented in [15].

Data-based approaches are preferable instead in case the system dynamics is not precisely known and/or when the system input-output physical relations are too complex [16]. Currently, thanks to the advances in ICT technologies in both hardware and software, substantial amounts of data are available for continuous monitoring and FD of engineering systems. This generated a great interest in FD techniques capable of handling large amounts of data. In this context, the most established and widespread techniques are Principal Component Analysis (PCA), Independent Component Analysis (ICA), Partial Least Squares (PLS), Fisher Discriminant Analysis (FDA), the Subspace-Aided approach (SAP), as well as their recent advancements [17]. Remaining in the data-based domain, FD diagnostic systems based on neural networks [18], computational intelligence [19], and machine learning [20,21] have attracted substantial interest in very recent years. Specifically, in the aviation and flight control systems community, there is abundant technical literature that clearly shows that the main reason for catastrophic accidents can be attributed, ultimately, to failures and/or malfunction of sensors, control surfaces, or components of the propulsion system, as reported in [22,23]. This explains the critical need for on-board FD systems able to promptly detect and identify the faulty components and, next, to enable a predetermined online failure recovery strategy [24]. Typically, hardware redundancy (featuring multiple sensors or actuators with the same function) and simple majority voting schemes are used to cope with FD. Although these methods are widespread, there is an increasing number of applications where the additional cost, weight, and size of the components are major constraints, such as small and autonomous flying vehicles. For these applications, AR-based FD methods are appealing approaches to increase flight safety. An excellent review on the state-of-the-art of AR-based FD methods in aerospace systems can be found in [25].

Regardless of the approach, the performance of an FD system is strictly related to the level of modeling uncertainty and measurement noise, whose presence implies that in fault-free conditions, the residual signals might significantly deviate statistically from zero. This entails that residuals must be robust with respect to uncertainties where the robustness of a residual is defined as the degree of sensitivity to faults compared to the sensitivity to uncertainty and noise [26]. Robustness issues become evident when dealing with experimental flight data [27,28,29,30,31]. Indeed, experimental data are affected by several additional sources of uncertainty such as, but not limited to, signal synchronization and quantization errors and imperfect positioning and calibration of the sensors [32,33]. Therefore, a fundamental step before the deployment of an FD scheme is its validation with actual flight data. Despite the relevance of these aspects in the mentioned studies, the problem of a detailed performance evaluation and comparison of Fault Isolation (FI) and Fault Estimation (FE) schemes using multi-flight data has been rarely fully addressed.

Building on the last considerations, the present study is entirely devoted to the setup and the quantitative comparison of well-known FI and FE methods using experimental sensor flight data. Specifically, different consolidated data-driven FI and FE schemes are applied to evaluate their performance on a set of 14 primary sensors of a semi-autonomous Tecnam P92 aircraft [34].

In the effort, experimental linear regression models of the sensors’ response are identified from correlated measurements using multivariate linear regression techniques [3]. The identified models provide a natural set of primary residuals; however, the faults’ directional properties are often not optimized to be used for sensor FI [35,36]. For this reason, enhanced residuals are derived by applying a linear transformation that allows better separation between the fault directions. The approach proposed in [37] that optimizes fault directions separation taking also into account experimental modeling errors is herein implemented.

Sensor FI is then performed using two different approaches. The first approach associates the fault with the sensor providing the smaller directional distance compared to the sensor fault directions [35]; the second approach, instead, is based on the reconstruction-based method proposed in [38].

A comprehensive (offline) analysis based on multi-flight data is performed to derive quantitative Fault Isolation (FI) and Fault Estimation (FE) performance as a function of the fault amplitudes.

Next, another study is performed to assess online sensor FI performance by monitoring the temporal history of the residuals and, using this information, to increase or decrease the belief in a fault hypothesis over time. For this purpose, a bank of recursive Bayesian filters is designed to infer in-flight sensors’ fault probabilities. During online operation, a fault is declared when a fault probability reaches a defined threshold. This allows the computation of FI delays. The accumulated evidence in the probabilistic filters allows more reliable fault isolation, decreasing the false alarm rate at the expense of a small and acceptable delay in the diagnosis.

A detailed evaluation of offline and online FD performance is presented for failures on the air data sensors, including the True Air Speed (TAS, TaS), the Angle of Attack (AoA, α), and the Angle of Sideslip (AoS, β).

The present study is application oriented; therefore, the main contribution is not the development of a novel FD technique, but, rather, the design of consolidated data-driven sensor FD schemes and the quantitative evolution of fundamental quantities such as the fault isolation percentage, fault reconstruction accuracy, and in-flight fault isolation delays using multi-flight validation data. Therefore, the study provides a clear picture of the required design effort along with the achievable performance using consolidated FD schemes.

The paper is organized as follows. Section 2 introduces linear regression models for sensors’ fault diagnosis; Section 3 introduces the FI and FE methods based on primary residuals, while Section 4 introduces the FI and FE methods based on transformed residuals. Section 5 introduces the Bayesian filters for online FI. Section 6 describes the multi-flight data, while Section 7 deals with the identification of experimental models. FI and FE results on validation data are discussed in Section 8 and Section 9. Concluding and summary remarks are provided in Section 10.

## 2. Models for Sensor Fault Diagnosis

In this study, a set of $n_{x}$ potentially faulty sensors is considered. The corresponding signals are concatenated in a vector $x\left(k\right)\in R^{n_{x}}$; $u\left(k\right)\in R^{n_{u}}$ is another vector of $n_{u}$ signals functionally correlated with x(k) such as control inputs, set-points, and other sensor measurements (assumed not to be faulty). It is assumed that in fault-free conditions, the sensor measurements x(k) can be expressed in linear regression form as a function of x(k) and u(k), that is:

(1) xi(k)=∑j=1j≠inxwxi,jxj(k)+∑j=1nuwui,juj(k)+Δi(k)i=1...nx

where $w_{xi,j}$ and $w_{ui,j}$ are the coefficients of the linear combination and $\Delta_{i}\left(k\right)$ characterizes modeling nonlinearity, uncertainty, and measurement noise concerning the *i*-th sensor. For simplicity, the linear models in (1) are rearranged as:

(2) $$x_{i}\left(k\right)=w_{xi}x\left(k\right)+w_{ui}u\left(k\right)+\Delta_{i}\left(k\right)\;i=1...n_{x}$$

where $w_{xi}=\left[w_{xi,1},...,w_{xi,i-1},0,w_{xi,i+1},...,w_{xi,n_{x}}\right]\in R^{n_{x}}$ and $w_{ui}=\left[w_{ui,1},...,w_{ui,n_{u}}\right]\in R^{n_{u}}$. Putting the above $n_{x}$ equations together, we get the following vector expression:

(3) $$x\left(k\right)=W_{x}x\left(k\right)+W_{u}u\left(k\right)+\Delta \left(k\right)$$

where $W_{x}=\left[w_{x1};...;w_{xn_{x}}\right]\in R^{n_{x}\times n_{x}}$ and $W_{u}=\left[w_{u1};...;w_{un_{u}}\right]\in R^{n_{x}\times n_{u}}$ are constant matrices to be estimated from data (as discussed in Section 7). Model (3) has been widely used in the literature for estimating a sensor signal as a function of other correlated measurements, as shown, for instance, in [35,39,40,41,42]. The linear terms in (3) provide a linear estimation of the signals that are defined as:

(4) $$\hat{x}\left(k\right)=W_{x}x\left(k\right)+W_{u}u\left(k\right).$$

The consistency of the measurements x(k) are monitored through the vector of the primary residuals $r_{0}\left(k\right)\in R^{n_{x}}$ that is defined as follows:

(5) $$r_{0}\left(k\right)=x\left(k\right)-\hat{x}\left(k\right).$$

Substituting (4) into (5) leads to:

(6) $$\begin{array}{c} r_{0}\left(k\right)=\left(I-W_{x}\right)x\left(k\right)-W_{u}u\left(k\right)\\ \;\;=Wx\left(k\right)-W_{u}u\left(k\right) \end{array}$$

where $I\in R^{n_{x}\times n_{x}}$ is the identity matrix and $W=I-W_{x}$. Using again (3) and (4) in (5), it is immediate to verify that:

(7) $$r_{0}\left(k\right)=\Delta \left(k\right)$$

In other words, in fault-free conditions, the vector of the primary residuals is equal to the modeling error.

### Sensor Fault Modeling

In this study, an additive (single) failure $f_{i}\left(k\right)$ on a generic (*i*-th) sensor of x(k) is considered. Without any loss of generality, the failure can affect every single sensor. In the presence of a sensor failure, the vector x(k) is replaced by its “faulty version”, that is:

(8) $$x\left(k\right)\leftarrow x\left(k\right)+\varepsilon_{i}f_{i}\left(k\right)$$

where $\varepsilon_{i}\in R^{n_{x}}$ is the *i*-th column of the identity matrix I. $f_{i}\left(k\right)$ is an arbitrary scalar, a function of time, modeling the fault shape; thus, $f_{i}\left(k\right)$ is zero before the fault occurrence and different from zero, starting from the “fault time instant” $k_{f}$. In this study, we consider a step fault:

(9) $$f_{i}\left(k\right)=\left\{\begin{array}{c} 0\;\;t<k_{f}\\ A_{i}\;t\geq k_{f} \end{array}\right.i=1...n_{x}$$

where $A_{i}$ is the fault amplitude associated with the *i*-th sensor.

## 3. Sensor FI and FE Based on Primary Residuals

The first FI scheme is derived directly from the analysis of the primary residual vector $r_{0}\left(k\right)$ in (6) in the presence of a sensor fault. Substituting the faulty vector (8) in (6) leads to the faulty residual:

(10) $$\begin{array}{c} r\left(k\right)=W\left[x\left(k\right)+\varepsilon_{i}f_{i}\left(k\right)\right]-W_{u}u\left(k\right)\\ \;\;=r_{0}\left(k\right)+W\left[\varepsilon_{i}f_{i}\left(k\right)\right]. \end{array}$$

Next, substituting in (10) the conditions (7) results in:

(11) $$r\left(k\right)=\Delta \left(k\right)+w_{i}f_{i}\left(k\right)$$

where the vector $w_{i}\in R^{n_{x}}$ is the *i*-th *column* of the matrix W. Vector $w_{i}$ is known as the “fault signature” or the “fault direction” associated with the sensor fault $f_{i}\left(k\right)$. In the theoretical case of zero modeling error in (11), this results in:

(12) $$r\left(k\right)=w_{i}f_{i}\left(k\right).$$

In other words, the vector r(k) is exactly parallel to vector $w_{i}$. This directional information can be used for sensor fault isolation purposes.

### 3.1. Mahalanobis Distance-Based FI and FE

This technique exploits the directional property of the $n_{x}$ primary residuals in (12) to formulate a sensor FI hypothesis [43]. Specifically, in the presence of a fault $f_{i}\left(k\right)$, Equation (12) implies that the residual vector r(k) assumes a specific known direction in the residual space $R^{n_{x}}$ given by the *i*-th column vector $w_{i}$ of matrix W. By taking advantage of this fault directional property, it is possible to identify the faulty sensor by comparing the direction of the current residual vector r(k) with the known $n_{x}$ directions $w_{i}$ and assigning the fault to the sensor whose direction is the closest to the r(k) direction. In practice, this FI logic is implemented as follows. As the first step, in order to be independent of the fault amplitude, both the residual r(k) and the sensor fault directions are normalized to a unity norm. Specifically, at each instant *k*, the normalized residual ($\left|\right|\bar{r}\left(k\right)|{|}_{2}=1$) is computed as $\bar{r}\left(k\right)=r\left(k\right)/{\left[\left(r^{T}\left(k\right)r\left(k\right)\right)\right]}^{0.5}$, while the normalized fault directions ($\left|\right|{\bar{w}}_{i}\left(k\right)|{|}_{2}=1\;\;i=1...n_{x}$) are defined as ${\bar{w}}_{i}=w_{i}/{\left[\left(w_{i}^{T}w_{i}\right)\right]}^{0.5}$. Next, since the faults can have either a positive or a negative amplitude, it was deemed necessary to introduce negative normalized fault directions ($-{\bar{w}}_{i}\;i=1...n_{x}$), implying that the overall number of fault directions is $2n_{x}$. The distance between the normalized residual $\bar{r}\left(k\right)$ and the normalized fault directions ${\bar{w}}_{i}$ can be measured using different norms such as the Euclidean distance, the angular distance, or the Mahalanobis distance [44,45]. In this study, we selected the Mahalanobis distance since this approach allows accounting for modeling error information derived from experimental data. The error matrix $G\left(k\right)\in R^{n_{x}\times 2n_{x}}$ of the differences between the normalized residual $\bar{r}\left(k\right)$ and the normalized fault directions is defined as:

(13) $$G\left(k\right)=[\bar{r}\left(k\right)-{\bar{w}}_{1},\;...,\bar{r}\left(k\right)-{\bar{w}}_{n_{x}},\;\;\bar{r}\left(k\right)+{\bar{w}}_{1}\;,...\;,\bar{r}\left(k\right)+{\bar{w}}_{n_{x}}]$$

where the first $n_{x}$ columns are associated with positive faults and the last $n_{x}$ with negative faults. Next, the Mahalanobis distance associated with the columns $g_{j}\in R^{n_{x}}$ of G(k) is defined as:

(14) $$e_{j}\left(k\right)=\sqrt{g_{j}^{T}S_{r}^{-1}g_{j}}\;j=1...2n_{x}$$

where $S_{r}\in R^{n_{x}\times n_{x}}$ is the covariance matrix of the residuals (for its computation, see Note 1). At each sample time instant *k* (following a fault detection), the FI is performed associating the fault with the sensor leading to the minimum value of the distances in (14). That is, defining $j_{m}\left(k\right)$ as:

(15) $$j_{m}\left(k\right)=\underset{j=1...2n_{x}}{argmin}\left(e_{j}\left(k\right)\right)$$

then the faulty sensor index is identified as follows:

(16) $$i_{F}\left(k\right)=\left\{\begin{array}{c} j_{m}\left(k\right)\;\;if\;1\leq j_{m}\left(k\right)\;\leq n_{x}\\ j_{m}\left(k\right)-n_{x}\;if\;n_{x}+1\leq j_{m}\left(k\right)\;\leq 2n_{x} \end{array}\right..$$

The amplitude of the fault is positive for $1\leq j_{m}\left(k\right)\;\leq n_{x}$, and it is negative for $n_{x}+1\leq j_{m}\left(k\right)\;\leq 2n_{x}$. Once a fault has been associated with the sensor $i_{F}\left(k\right)$, its amplitude can be directly estimated considering the $i_{F}$-th component $r_{iF}\left(k\right)$ of the primary residual vector r(k). In fact, from (11), this results in:

(17) $$r_{iF}\left(k\right)=\Delta_{iF}\left(k\right)+w{}_{\left(iF,iF\right)}f_{iF}\left(k\right).$$

Next, since by construction, $w{}_{\left(iF,iF\right)}=1\;\forall i_{F}\left(k\right)$, this results in:

(18) $$r_{iF}\left(k\right)=\Delta_{iF}\left(k\right)+f_{iF}\left(k\right).$$

In other words, $r_{iF}\left(k\right)$ provides a direct (noisy) estimation of the fault amplitude ${\hat{A}}_{i}\left(k\right)={\hat{f}}_{iF}\left(k\right)$ defined in (9), that is:

(19) $$r_{iF}\left(k\right)={\hat{f}}_{iF}\left(k\right)={\hat{A}}_{i}\left(k\right).$$

### 3.2. Reconstruction-Based FI and FE

Another approach for FI and FE is the well-known reconstruction-based method [38,46,47]. In this approach, for each fault direction $w_{i}$, the fault amplitude, at time instant *k*, is estimated by computing the fault amplitude $f_{i}\left(k\right)$ that minimizes the norm-two of the reconstruction errors defined as:

(20) $$e_{i}\left(k\right)={\left[{\left(r\left(k\right)-w_{i}f_{i}\left(k\right)\right)}^{T}\left(r\left(k\right)-w_{i}f_{i}\left(k\right)\right)\right]}^{0.5}\;i=1...n_{x}.$$

The optimal values for $f_{i}\left(k\right)$ are derived by computing the minimum of the quadratic form (20) with respect to the scalar $f_{i}\left(k\right)$. This leads to the closed-form solution:

(21) $${\hat{f}}_{i}\left(k\right)=\frac{w_{i}^{T}r\left(k\right)}{w_{i}^{T}w_{i}}\;i=1...n_{x}.$$

It is observed that the fault amplitude estimation ${\hat{f}}_{i}\left(k\right)$ provided by (21) can be either positive or negative, implying that the reconstruction-based approach handles both positive and negative faults. The faulty sensor is then isolated associating the fault with the sensor leading to the minimum of the “reconstructed” errors achieved substituting the fault estimates (21) in (20). Therefore, the sensor fault index is:

(22) $$i_{F}\left(k\right)=\underset{j=1...n_{x}}{argmin}\left(e_{j}\left(k\right)\right).$$

Similar to (19), the estimated fault amplitude is:

(23) $${\hat{A}}_{i}\left(k\right)=r_{iF}\left(k\right).$$

Note 1: The residual noise covariance matrix $S_{r}$ in (14) depends on the modeling uncertainty Δ(k) in (7), which is very difficult to characterize “a priori”. For this reason, $S_{r}$ was estimated from the experimental data. From (7), it is inferred that in fault-free conditions, the relationship $r_{0}\left(k\right)=\Delta \left(k\right)$ holds. This allows estimating $S_{r}$ as the sample covariance matrix of the residual vector inferred from experimental flight data in fault-free conditions. The experimental $S_{r}$ matrix is therefore:

(24) $${\hat{S}}_{r}=\mathrm{cov}\left\{r_{0}\left(k\right)\right\}=\mathrm{cov}\left\{\Delta (k)\right\}={\hat{S}}_{\Delta }\;k\in \mathrm{train}\_\mathrm{data}.$$

Note 2: In this effort, we assumed that faults originate from sensors in an additive fashion. Under this hypothesis, it is possible to isolate the faulty sensor by analyzing the fault direction of the residual signals as shown in Equation (9). It is worth emphasizing that primary residuals can also be affected by the occurrence of generic internal system faults that in turn produce a specific fault signature on the residuals. In the case of internal faults, it is necessary to reconstruct the roots of the fault symptoms in the subsystems that constitute a complex system in order to be able to isolate the faulty component. In the event an internal fault produces a signature identical to those of a sensor fault, they would not be distinguishable by our scheme. The isolation of internal faults requires the knowledge of the internal dynamics of the subsystems and its cause-effect relationships. This problem, although interesting, is beyond the scope of this study, which is limited to addressing the sensor fault isolation problem. An interesting review on the cause and fault propagation can be found in [48].

## 4. Sensor FI and FE Based on Transformed Residuals

Primary residuals are not optimized from an FI point of view. For this reason, they are usually processed by applying transformations to achieve suitable new fault directions that facilitate the FI. An extensive literature on transformed directional residuals techniques is available. One of the first applications is the the Beard–Jones filter [13], essentially a Luenberger observer whose gains are selected so that the directions of the residuals can be advantageously used to identify faulty sensors. In [49], taking advantage of the properties of un-observability subspaces, a set of residual transformations that are unaffected by all faults except one was proposed. In this context, the methodology outlined in [36] is also relevant, where the interaction between directional residuals and fault isolation properties was analyzed.

The FI methodology proposed in [37] was considered in this effort because it provides optimized robust performance considering the directional properties of the residual noise covariance matrix $S_{r}$. This feature is particularly important when dealing with experimental noisy data. In the approach in [37], a linear transformation of the primary residual vector r(k) is introduced to provide optimized performance with respect to noise immunity. A transformed residual $\rho \left(k\right)\in R^{n_{x}}$ with the same number of elements of r(k) is defined as:

(25) $$\rho \left(k\right)=W_{t}r\left(k\right)=W_{t}\left[Wx\left(k\right)-W_{u}u\left(k\right)\right]$$

where $W_{t}\in R^{n_{x}\times n_{x}}$ is the transformation matrix to be computed. Considering (25), the new fault directions associated with ρ(k) are the columns ${w_{\rho }}_{i}$ of the matrix:

(26) $$W_{\rho }=W_{t}W\;\in R^{n_{x}\times n_{x}}.$$

The covariance of ρ(k) is by definition:

(27) $$S_{\rho }=\mathrm{cov}\left\{W_{t}\;r\left(k\right)\right\}=\;W_{t}\;S_{r}\;{W_{t}}^{T}$$

where the matrix $W_{t}$ is computed applying the method proposed in [37]. Considering this approach, let $A\in R^{n_{x}\times n_{x}}$ and $B\in R^{n_{x}\times n_{x}}$ be two symmetric matrices. Assuming A positive definite, it can be shown that there exists a matrix $V\in R^{n_{x}\times n_{x}}$ such that:

(28) $$V^{T}A\;V=I,$$

(29) $$V^{T}\;B\;V=\Lambda =\mathrm{Diag}\left(\lambda_{1},\ldots ,\lambda_{n}\right)$$

where V and Λ are the solutions of the generalized eigenvalue problem. Specifically, the columns of V are the eigenvectors of the matrix $A^{-1}B$, while the columns of Λ are the corresponding eigenvalues. In the present study, setting $A=S_{r}$, $B=W\;W^{T}$, and $V^{T}=W_{t}$, Equations (28) and (29) become, respectively:

(30) $$W_{t}\;S_{r}\;{W_{t}}^{T}=I,$$

(31) $$W_{t}WW^{T}{W_{t}}^{T}=W_{\rho }{W_{\rho }}^{T}=\Lambda .$$

Relationship (30) implies that the noise covariance matrix of the transformed residuals in (27) is spherical, that is $S_{\rho }=I$. This property is critical since in the presence of a spherical symmetry noise, the optimal decision line between two fault directions is simply the bisector [50]. Thus, this property will be exploited for the design of the FI algorithm in Section 4.1.

As explained in Note 1, the $S_{r}$ matrix is estimated using the experimental ${\hat{S}}_{r}$ in (24), and the generalized eigenvalues Λ are derived using commercially available scientific software.

### 4.1. Transformed Residuals Based on FI and FE

Fault isolation based on the transformed residual ρ(k) is performed using the same approaches used for the primary residuals in Section 3.1 and Section 3.2. Specifically, defining the new transformed residual directions ${w_{\rho }}_{i}\;i=1...n_{x}$ as the columns of the matrix $W_{\rho }$, the transformed error matrix $G_{\rho }\left(k\right)\in R^{n_{x}\times 2n_{x}}$ is defined as:

(32) $$G_{\rho }\left(k\right)=\left[\bar{\rho }\left(k\right)-{\bar{w}}_{\rho_{1}},...,\bar{\rho }\left(k\right)-{\bar{w}}_{\rho_{n_{x}}},\bar{\rho }\left(k\right)+{\bar{w}}_{\rho_{1}},...,\bar{\rho }\left(k\right)+{\bar{w}}_{\rho_{n_{x}}}\right]$$

where $\bar{\rho }\left(k\right)$ is the normalized transformed residual derived from ρ(k) and ${\bar{w}}_{\rho_{i}}\;i=1...n_{x}$ are the new normalized transformed fault directions derived from $w_{\rho_{i}}$. The distance errors associated with the columns $g_{\rho_{i}}$ of the matrix $G_{\rho }\left(k\right)$ are defined as:

(33) $${e_{\rho }}_{j}\left(k\right)=\sqrt{{g_{\rho_{j}}}^{T}g_{\rho_{j}\;}}\;j=1...2n_{x}.$$

The fault isolation index is determined by applying the same technique introduced in Section 3.1, that is:

(34) $$j_{m}\left(k\right)=\underset{j=1...2n_{x}}{argmin}\left(e_{\rho_{j}}\left(k\right)\right).$$

It is observed that the decision method (34) based on the Euclidean distances (33) is equivalent to treating as the decision line the bisectors between the directions $g_{\rho_{i}}$ in (32), which, in the case of spherical noise ($S_{r}=I$), are also optimal for testing fault isolation [50].

The faulty sensor $i_{F}\left(k\right)$ is isolated applying (16) to $j_{m}\left(k\right)$ derived from (34). Finally, the fault amplitude is estimated using again (19), that is:

(35) $$r_{iF}\left(k\right)={\hat{A}}_{i}\left(k\right)=\hat{f}{}_{iF}\left(k\right).$$

Note 3: In [37], it was shown that the best performances are achieved in the case that the transformation matrix $W_{t}$ leads to the diagonalization, not only of the noise covariance matrix $S_{\rho }$, but also of the transformed fault direction matrix $W_{\rho }$. This may be possible only if the number of residuals is larger than the number of sensors. Alternatively, approximated robust FI methods based on optimality concepts as those in [35,51] can be applied.

### 4.2. Transformed Residuals with Reconstruction-Based FI and FE

Consistent with the approach of Section 3.2, the reconstruction error for transformed residuals is defined as:

(36) $$e_{\rho_{i}}\left(k\right)={\left[{\left(\rho \left(k\right)-w_{\rho_{i}}f_{i}\left(k\right)\right)}^{T}\left(\rho \left(k\right)-w_{\rho_{i}}f_{i}\left(k\right)\right)\right]}^{0.5}\;i=1...n_{x}$$

while the reconstructed fault amplitude is:

(37) $${\hat{f}}_{i}\left(k\right)=\frac{w_{\rho_{i}}^{T}\rho \left(k\right)}{w_{\rho_{i}}^{T}w_{\rho_{i}}}\;i=1...n_{x}.$$

Fault isolation is again performed by computing the minimum of the errors in (36); in other words, the fault isolation index is:

(38) $$i_{F}\left(k\right)=\underset{j=1...n_{x}}{argmin}\left(e_{\rho_{j}}\left(k\right)\right).$$

Finally, similar to (23), the estimated fault is:

(39) $${\hat{A}}_{i}\left(k\right)=\hat{f}{}_{iF}\left(k\right).$$

## 5. Bayesian Filtering for Online Fault Isolation

The FI approaches described in Section 3 and Section 4 are based on a decision method that isolates the faulty sensor as the one providing the minimum of a specific error measure inferred from the residual. This FI logic is based only on information at the current sample time *k* and does not take into account the history of the residuals in the previous instants. On the other side, this information is useful for increasing or decreasing the belief of the FI decision over time.

In this paper, we propose a Bayesian Filter (BF) approach for managing the stream of information coming from sensors’ measurements. Specifically, we implemented the so-called “discrete Bayes filter” proposed in [52]. This filter is essentially a recursive algorithm used to estimate the distribution of a discrete probability function. This type of filter was selected because it builds on a very solid and comprehensible theoretical background; an introduction to the Bayesian decision theory can be found in [53]. In the present study, the estimated distribution models the probability (belief) that a generic sensor is faulty. The BF infers sensor fault probabilities by processing recursively the error information $e_{i}\left(k\right)\;i=1...n_{x}$ defined in (14), (20), (33) and (36) for the different methods.

Two possible operational statuses are assumed for the sensors. State $F_{i}$ indicates the events for which the *i*-th sensor is faulty, while state $N_{i}$ the event for which it is not faulty. Let $p\left(e_{i}\left(k\right)|F_{i}\left(k\right)\right)$ be the likelihood function representing the probability of observing an error $e_{i}\left(k\right)$ given that the *i*-th sensor is faulty; a similar definition holds for $p\left(e_{i}\left(k\right)|N_{i}\left(k\right)\right)$. According to Bayes’ theorem, the posterior fault probabilities $p\left(F_{i}\left(k\right)|e_{i}\left(k\right)\right)$ are given by:

(40) $$\begin{array}{cc} p\left(F_{i}\left(k\right)|e_{i}\left(k\right)\right) & =\frac{p\left(e_{i}\left(k\right)|F_{i}\left(k\right)\right)p\left(F_{i}\left(k\right)\right)}{p\left(e_{i}\left(k\right)|F_{i}\left(k\right)\right)p\left(F_{i}\left(k\right)\right)+p\left(e_{i}\left(k\right)|N_{i}\left(k\right)\right)p\left(N_{i}\left(k\right)\right)}\\ p\left(N_{i}\left(k\right)|e_{i}\left(k\right)\right) & =1-p\left(F_{i}\left(k\right)|e_{i}\left(k\right)\right) \end{array}\;i=1...n_{x}$$

where $p\left(F_{i}\left(k\right)\right)$ and $p\left(N_{i}\left(k\right)\right)$ are the a priori fault and non-fault probabilities, respectively, for sensor *i*. A key step in the above inference mechanism is the definition of the likelihood functions $p\left(e_{i}\left(k\right)|F_{i}\left(k\right)\right)$. These have to be designed to model the probability of experiencing a distance error $e_{i}\left(k\right)$ given that the *i*-th sensor is faulty; therefore, their distribution needs to be maximum for $e_{i}\left(k\right)=0$ and should decrease as $e_{i}\left(k\right)$ increases. Similar reasoning applies to $p\left(e_{i}\left(k\right)|N_{i}\left(k\right)\right)$. This behavior is captured by the following likelihood functions [54,55,56,57]:

(41) $$\begin{array}{c} p\left(e_{i}\left(k\right)|F_{i}\left(k\right)\right)=e^{-\alpha_{i}e_{i}\left(k\right)},\\ p\left(e_{i}\left(k\right)|N_{i}\left(k\right)\right)=e^{-\alpha_{i}{\left[e_{i}\left(k\right)\right]}^{-1}} \end{array}$$

where the parameters $\alpha_{i}$ are used to regulate the shape of the probability functions. At each time step, following the computation of the posterior probabilities (40), the a priori probabilities are updated recursively using:

(42) $$\begin{array}{c} p\left(F_{i}\left(k+1\right)\right)=p\left(F_{i}\left(k\right)|e_{i}\left(k\right)\right),\\ p\left(N_{i}\left(k+1\right)\right)=p\left(N_{i}\left(k\right)|e_{i}\left(k\right)\right) \end{array}$$

to propagate the current probability information to the next time step. In this study, the probabilistic filters are activated immediately after a fault is detected at $k=k_{f}$. At instant $k_{f}$, it is assumed that all the $n_{x}$ sensors have the identical probability to be faulty; in other words, the filters are initialized with:

(43) $$\begin{array}{cc} p\left(F_{i}\left(k{}_{f}\right)\right) & =1/n_{x}\\ p\left(N_{i}\left(k{}_{f}\right)\right) & =1-p\left(F_{i}\left(k{}_{f}\right)\right) \end{array}\;i=1\ldots n_{x}.$$

### 5.1. Probabilistic (Online) Fault Isolation Method

The $n_{x}$ recursive BF probabilities are employed for online FI, which is associated with in-flight conditions. Following a fault detection at $k=k_{f}$, the fault is assigned to the sensor whose fault probability $p\left(F_{i}\left(k\right)|e_{i}\left(k\right)\right)$ first reaches a defined threshold. If $p\left(F_{i}\left(k\right)|e_{i}\left(k\right)\right)$ exceeds the threshold, the FI scheme has “good confidence” that a fault has occurred on the *i*-th sensor. Therefore, a failure on that sensor is declared. In this study, the threshold was empirically set at 0.7 (70%); see Note 4.

### 5.2. Probability Function Tuning

The performance of the BFs depends strictly on the shape of the probability functions in (42), which, in turn, depend on the values of the parameters $\alpha_{i}$. These values have to be carefully selected to make the filters sensitive to faults while limiting false alarms. Interestingly, a significant difference in the tuning of the filters was found for the distance-based and reconstruction-based techniques. This difference is essential because, while the first method operates with normalized residuals, the second operates with non-normalized residuals.

#### 5.2.1. Distance-Based Methods’ Tuning

For the distance-based techniques, it was relatively simple to calibrate $\alpha_{i}$ to limit false alarms. These techniques being based on normalized residuals ($|\bar{r}\left(k\right)|=1$, $|\bar{\rho }\left(k\right)|=1$), the $\alpha_{i}$ values were inferred from the mean value (in fault-free conditions) of the distance between the normalized residual and normalized fault directions. Then, for distances $e_{i}\left(k\right)$ smaller than the mean value of the distance, the $\alpha_{i}$ values of $p\left(F_{i}\left(k\right)|e_{i}\left(k\right)\right)$ in (41) are tuned to be larger than 0.5, so that the BF increases the fault belief with respect to the previous step. Conversely, for larger distances, $p\left(F_{i}\left(k\right)|e_{i}\left(k\right)\right)$ is lower than 0.5, resulting in a decrease in the belief. This tuning method was found to be effective since the distance $e_{i}\left(k\right)$ (independent of the fault size) is always contained in the hyper-ellipsoid (14).

#### 5.2.2. Reconstruction-Based Methods’ Tuning

For reconstruction-based techniques, the application of the above calibration technique is not possible. This is due to the fact that the errors $e_{i}\left(k\right)$ in (20) derive from non-normalized residuals (r(k) and ρ(k)), and therefore, their values are proportional to the fault amplitude. Thus, the selection of $\alpha_{i}$ values that are suitable for any fault amplitude is not trivial. To overcome the problem, we opted for a “forced” normalization of the errors $e_{i}\left(k\right)$ in (20) to be used as the input of the BFs. The following normalization method was applied. At each sample instant *k*, the 14 errors $e_{i}\left(k\right)$ are ranked so that $e_{rank_{1}}\left(k\right)\leq e_{rank_{2}}\left(k\right)\leq e_{rank_{3}}\left(k\right)...\leq e_{rank_{14}}\left(k\right)$; next, normalized errors ${\bar{e}}_{i}\left(k\right)$ are defined as ${\bar{e}}_{i}\left(k\right)=e_{i}\left(k\right)/e_{rank_{2}}\left(k\right)$. Through this scheme, the new ${\bar{e}}_{i}\left(k\right)$ are such that one normalized error is always less than one; another is exactly equal to one, while all the remaining normalized errors are larger than one. Using the normalized ${\bar{e}}_{i}\left(k\right)$, it is possible to define a simple tuning strategy for $\alpha_{i}$. Specifically, $\alpha_{i}$ are tuned such that the fault belief increases for the (nearest) sensor having ${\bar{e}}_{i}\left(k\right)\leq 1$, remains unchanged for the sensor having ${\bar{e}}_{i}\left(k\right)=1$, and decreases for all the remaining sensors. It is immediate to verify that $\alpha_{i}=\ln2$ guarantees this behavior. The normalized errors ${\bar{e}}_{i}\left(k\right)$ are then used in the BFs (40)–(43) (in place of $e_{i}\left(k\right)$) for the reconstruction based methods.

Note 4: An important aspect in the design of a fault diagnosis scheme is the definition of the threshold values to be used for fault detection and isolation. Clearly, the values of these thresholds have a direct impact on the missed alarm rate and false alarm rate. Although several methods have been introduced and tested to compute optimized thresholds [58,59], most of the detection methods require a priori knowledge of the signal distribution, changed parameters, and the change amplitude (CUSUM, likelihood ratio test, etc.). Furthermore, these methods assume that modeling errors are Independent and Identically Distributed (IID) random variables. Unfortunately, in our study, we found that the above-mentioned assumptions are not satisfied by experimental residuals and that the application of theoretical thresholds produces very conservative results lacking any practical utility. For this reason, these values were set empirically by trial and error.

Note 5: Since FI algorithms operates in real time, important factors are their computational and memory space requirements. Given that the transformation of fault signatures is performed offline, techniques based on transformed and primary residuals require exactly the same memory space for the storage of the models. As for the computational cost, in reconstruction-based techniques, the computational complexity is $O(n^{2})$, and the memory space required is $3n_{x}$ ($n_{x}$ to store residuals, $n_{x}$ to store estimated fault amplitudes, and $n_{x}$ to store errors). In distance-based techniques, the computational complexity, instead, depends on the operations requested to compute the errors in (20) or (36). For the technique with primary residuals, the complexity is $O(n^{3})$ because the matrix product involving ${Sr}^{-1}$ in (14), while in the case of transformed residuals, it is equal to $O(n^{2})$. The memory space required is quantifiable in $6n_{x}$ ($n_{x}$ to store the residuals, $n_{x}$ to store the normalized residuals, $2n_{x}$ to store G(k) (or $G_{\rho }\left(k\right)$), and $2n_{x}$ to store the errors). The overall results are summarized in Table 1. The Bayesian filtering introduces a computational complexity O(n) to calculate the posterior fault probabilities and requires a memory space $4n_{x}$ ($2n_{x}$ to store the likelihood probabilities and $2n_{x}$ to store the posterior fault probabilities).

## 6. Aircraft and Flight Data

**Figure 1:**
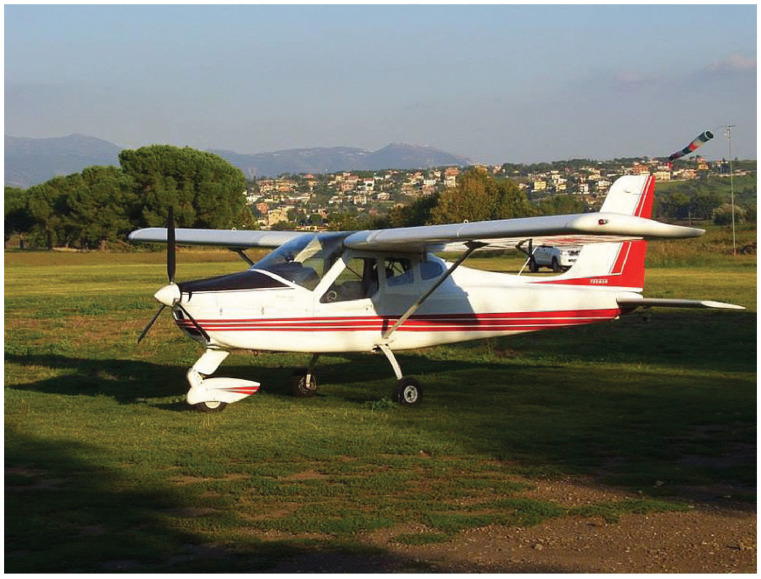
The semi-autonomous Tecnam P92 aircraft.

The considered FI and FE techniques were designed and validated using sensor flight data from a Tecnam P92 aircraft, shown in Figure 1. The aircraft mass is approximately 600 kg, and the propulsion is provided by a 74 kW Rotax 912 ULS with a two-blade fixed-pitch propeller, for a maximum cruise speed of 219 km/h and an operational ceiling of 4200 m. Data were acquired in semi-autonomous mode; in other words, the aircraft was manually flown by a pilot during the take-off and landing phase, while flown autonomously in cruise flight conditions. A set of nine flight datasets was considered in this study, of which five flights (with a total length of 2 h and 20 min) were used for the design of the FI and FE schemes, while the remaining four (with a total length of 2 h and 5 min) were used for validation purposes. These proprietary flight data previously acquired as part of an internal industrial research study for aircraft certification purposes were provided courtesy of Tecnam corporation. The data sampling time was set at 0.1 s. The study did not consider data associated with take-off, initial climb, final descent, and landing phases for the specific reason that in those flight conditions, the aerodynamic behavior of the aircraft is quite different with respect to cruise conditions due to the deployment of flaps. The use of multi-flight data for the training and the validation tasks is extremely important because, as for all data-driven techniques, the reliability of the results strongly depends on the completeness of the data, their spectral richness, and the coverage of all operating conditions. A total of 20 sensors was considered in this study, and they are listed in Table 2. The FI schemes were designed for the set $x_{0}$ of the 14 sensors indicated in Table 2 (the x(k) signals in (3), while the six actuation signals $u_{0}$ were the control deflections and engine commands (the u(k) signals in (3).

### Data Normalization

The experimental data listed in Table 2 have quite different ranges and orders of magnitude; this issue suggested using a data normalization to zero mean and unit standard deviation.

The presence of regressor signals with very different operative ranges could cause the exclusion of the informative regressors with lower power from the model. This problem is avoided through normalization, which makes all signals with zero mean and unit variance. For each a signal, the normalization was performed using:

(44) $$z_{i}\left(k\right)=\frac{z_{0_{i}}\left(k\right)-\mu_{z_{0_{i}}}}{\sigma_{z_{0_{i}}}}\;\;i=1\ldots \left(n_{x}+n_{u}\right)$$

where $z_{0_{i}}\left(k\right)$ is the *i*-th non-normalized signal ($x_{0_{i}}\left(k\right)$ or $u_{0_{i}}\left(k\right)$); $\mu_{z0_{i}}\left(k\right)$ is its mean, and $\sigma_{z_{0_{i}}}$ is its standard deviation. Since the $z_{i}\left(k\right)$ signals are normalized, this implies that faults in (8) are associated with normalized signals; therefore, the fault amplitude estimations provided by (19), (23), (37) and (39) must be de-normalized to recover the actual fault amplitude. This can be achieved inverting (44), so that, assuming ${\hat{f}}_{i}\left(k\right)$ to be the normalized fault reconstructed through (19), (23), (37) and (39), the actual amplitude of the fault is:

(45) $${\hat{f}}_{i_{den}}\left(k\right)=\sigma_{x_{0_{i}}}{\hat{f}}_{i}\left(k\right).$$

## 7. Experimental Models for Sensor FI

The fault isolation techniques introduced in Section 3 and Section 4 require the definition of linear multivariate models. Specifically, the definition of the matrices $W_{x}$ and $W_{u}$ associated with the $n_{x}=14$ linear models in (4) is required. For each one of the $n_{x}$ sensors, the model was identified separately using standard system identification techniques [3]. From (3), the resulting models are:

(46) $${\hat{x}}_{i}\left(k\right)=w_{x_{i}}\;x\left(k\right)+w_{u_{i}}\;u\left(k\right)\;\;\;i=1\ldots 14$$

where $w_{xi}\in R^{14}$ and $w_{ui}\in R^{6}$ are the *i*-th row of the matrices $W_{x}$ and $W_{u}$, respectively. Each of these 14 models depends on 20 potential regressors. It is common practice in model identification to select a subset of regressors that are critical for the estimation of the predicted output ${\hat{x}}_{i}\left(k\right)$. The regressors’ selection step was partially automated using the algorithm known as the “stepwise regressor selection method”. This is a well-known iterative data-driven algorithm for implementing a linear model by successively adding and/or removing regressors based on their statistical significance in a regression model [60]. In this effort, the stepwise selection was based on the training data using the ad-hoc procedure available in [61]. For each model, the selection of the best set of regressors was formulated through the analysis of the Root Mean Squared Error (RMSE) of the prediction error defined as:

(47) $$RMSE_{i}=\sqrt{mean\;\left[{\left(x_{i}\left(k\right)-{\hat{x}}_{i}\left(k\right)\right)}^{2}\right]}\;\;i=1...14.$$

**Figure 2:**
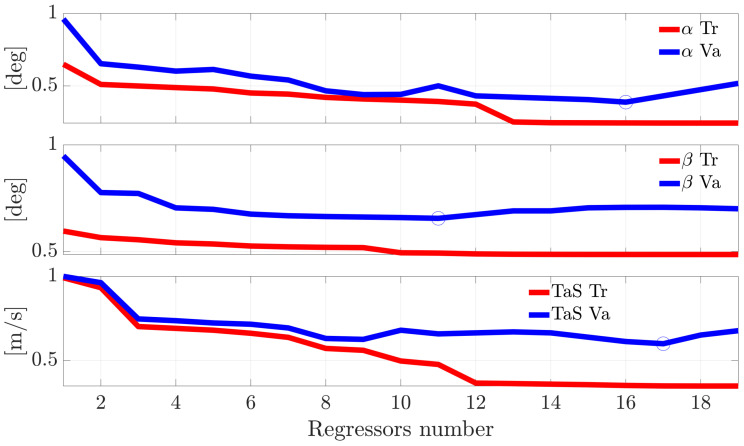
RMSE as function of the number of regressors in the model for the α(k), β(k), and TaS(k) sensors. Training (Tr) and Validation (Va) data.

Figure 2 shows the evolution of the RMSE for the models of the sensors α, β, and TaS evaluated both on the training and the validation flight data as a function of the number of regressors included in the model. As expected, while the training data result in a monotone decrease of the RMSE with the increase of the number of regressors in the model, for the validation data, the RMSE reaches a minimum; also, a larger number of regressors induces a decrease of the prediction accuracy. This is a typical example of the well-known modeling overfitting problem. Consequently, for each sensor, the best model was identified as the one that produced the minimum RMSE on the validation data set. The sets of regressors selected by this approach for the α, β, and TaS models are reported in Table 3. Without any loss of generality, this procedure was also applied to the other remaining 11 sensors. The number of regressors selected for the 14 models is reported in Table 4.

**Figure 3:**
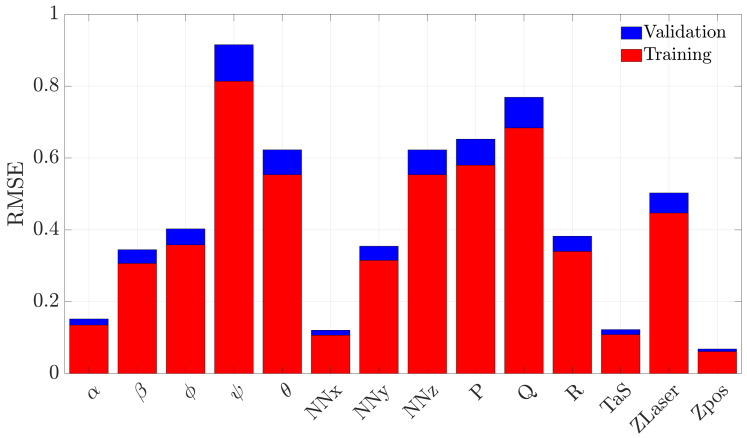
Comparison of the RMSE achieved in training and validation for the 14 linear regression models.

Figure 3 compares the RMSE for the 14 models produced by the (normalized) training and validation flight data. It is observed, for all models, that the training and the validation performance are comparable; this implies that the adopted regressor selection procedure is substantially correct and successfully avoids overfitting problems.

## 8. FI and FE Performance on the Validation Data (Offline Analysis)

For brevity purposes, only the results of the analysis relative to the air data sensors α(k), β(k), and TaS(k) are reported. However, it should be emphasized that the implemented schemes consider the entire set of $n_{x}=14$ sensors (residuals); in other words, the FI schemes isolate one among the 14 sensors.

Further, since the main purpose of this research is to compare the performance of the FI and FE techniques, the following analysis was performed assuming an “ideal” failure detection, i.e., the occurrence of a fault is detected as soon as it is injected into a generic time instant $k=k_{f}$. Clearly, in practice, fault detection is not instantaneous, and a fault detection delay is to be expected before the FI and FE algorithms are activated.

The overall evaluation of the FI and FE of the schemes was performed evaluating the average performance provided by the set of four validation flights (approximately 2.05 h) by injecting the faults in the first sample of each validation flight (kf=1). The analysis was then performed considering, for each sensor, 50 equally-spaced fault amplitudes $A_{i}$ in (9) in the range [$-A_{M_{i}}$; $+A_{M_{i}}$]. The maximum amplitudes $A_{M_{i}}$ (see Table 5) were selected empirically so that, for $A_{i}=A_{M_{i}}$, the FI algorithms correctly isolate the fault with a percentage greater than 80% (see Section 8.1).

The performance was analyzed using two specific metrics, that is the Fault Isolation Percentage (FIP) and the Relative Fault Reconstruction Error (RFRE). Both metrics are described below.

### 8.1. Fault Isolation Percentage

Considering a fault on the *i*-th sensor of amplitude $A_{i}$ in the *j*-th validation flight, the Fault Isolation Percentage (FIP) denoted by $I_{{\%}_{i}}$ is defined as the percent ratio over all the validation flights between the number of samples for which the fault is correctly attributed to the *i*-th sensor and the total number of samples:

(48) $$I_{{\%}_{i}}\left(A_{i}\right)=100\cdot \sum_{j=1}^{N_{val}}N_{OK_{j,i}}\left(A_{i}\right)/\sum_{j=1}^{N_{val}}N_{I_{j}}$$

where:

- $N_{val}$: number of validation flights.
- $N_{OK_{j,i}}\left(A_{i}\right)$: number of samples for which the fault isolation index $i_{F}\left(k\right)$ correctly isolates the fault on the *i*-th sensor, in validation flight *j*, for fault amplitude $A_{i}$.
- $N_{I_{j}}$: total number of samples in validation flight *j*.

**Figure 4:**
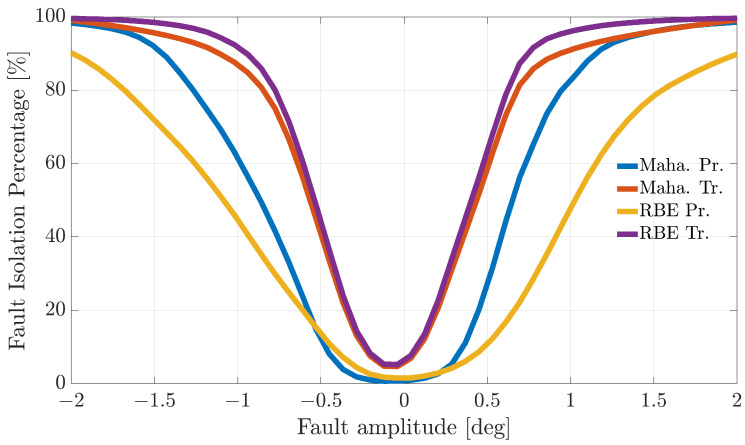
Fault isolation percentage for the α(k) sensor evaluated on the validation flights as a function of the fault amplitude.

**Figure 5:**
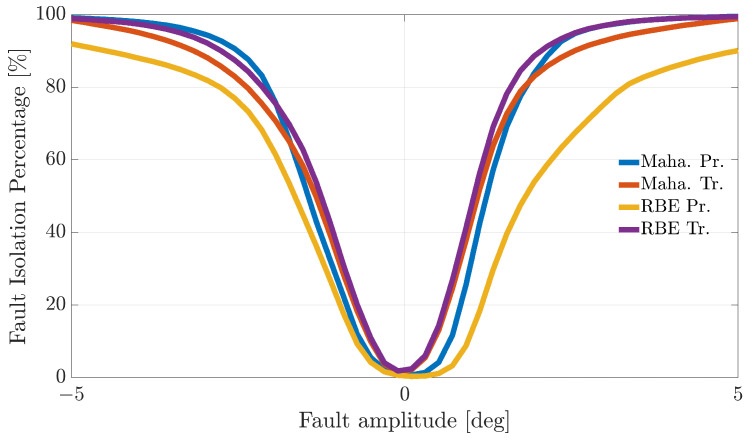
Fault isolation percentage for the β(k) sensor evaluated on the validation flights as a function of fault amplitude.

**Figure 6:**
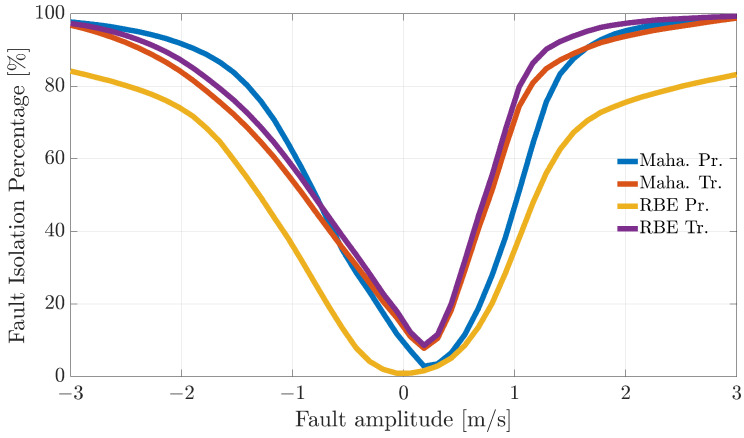
Fault isolation percentage for the TaS(k) sensor evaluated on the validation flights as a function of fault amplitude.

Figure 4, Figure 5 and Figure 6 show the FIP for the three sensors as a function of the fault amplitude computed using the $N_{val}=4$ validation flights for each of the considered techniques.

The analysis of the plots reveals that all the techniques can guarantee 100% correct fault isolation for large enough fault amplitudes for all the sensors. On the other side, small amplitude faults are often misinterpreted and misclassified. This is not surprising since small-amplitude faults have amplitudes similar to those of modeling errors, making the FI unreliable. The techniques Maha-Trand RBE-Tr based on transformed residuals provide better performance compared to the techniques based on primary residuals. This highlights the fact that the residual transformation based on the diagonalization of the noise covariance matrix is effective at improving FI performance.

### 8.2. Relative Fault Reconstruction Error

Considering a fault on the *i*-th sensor of amplitude $A_{i}$ in the *j*-th validation flight, the Relative Fault Reconstruction Error (RFRE) is defined as the percent mean relative amplitude reconstruction error, that is:

(49) $$E_{{\%}_{i}}\left(A_{i}\right)=100\cdot \sum_{j=1}^{N_{val}}\;\;\sum_{k\in S_{E_{j}}}^{}\left|\frac{{\hat{A}}_{i_{j}}\left(k\right)-A_{i}}{A_{i}}\right|/\sum_{j=1}^{N_{val}}N_{E_{j}}$$

where:

- $A_{i}$: amplitude of the fault on sensor *i*;
- ${\hat{A}}_{i_{j}}\left(k\right)$: amplitude of the reconstructed fault at sample time *k* for the validation flight *j*.
- $S_{E_{j}}$: set of samples in validation flight *j* where the fault is correctly attributed to the *i*-th sensor.
- $N_{E_{j}}$: number of samples in the set $S_{E_{j}}$.

**Figure 7:**
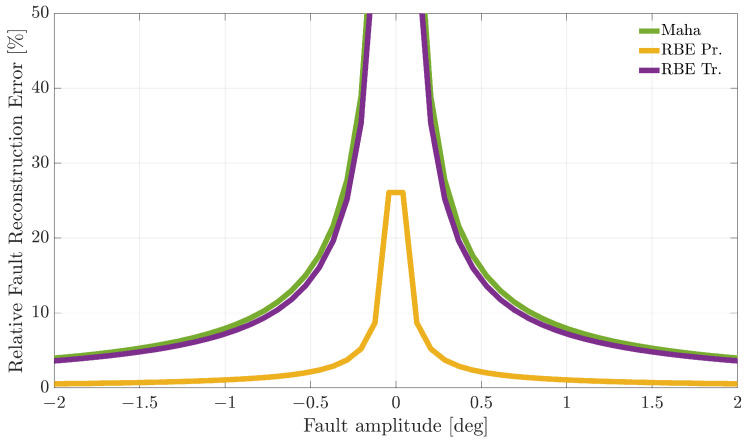
Relative fault reconstruction error for the α(k) sensor evaluated on the validation flights as a function of fault amplitude.

**Figure 8:**
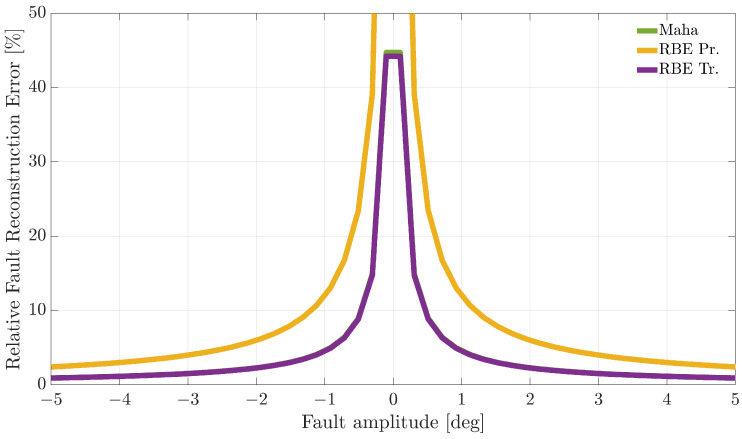
Relative fault reconstruction error for the β(k) sensor evaluated on the validation flights as a function of fault amplitude.

**Figure 9:**
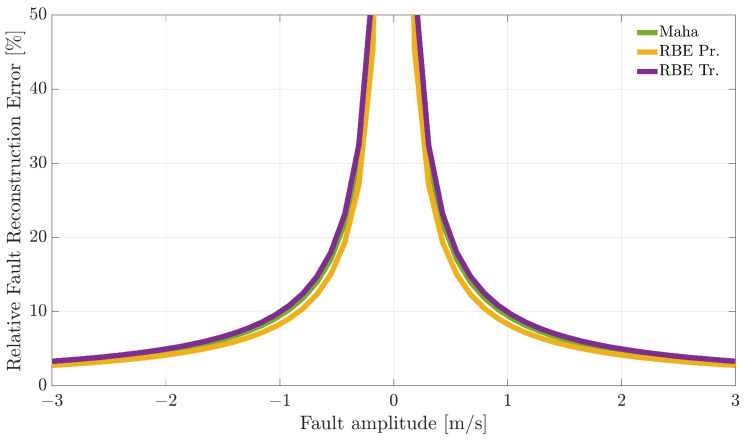
Relative fault reconstruction error for the TaS(k) sensor evaluated on the validation flights as a function of fault amplitude.

Figure 7, Figure 8 and Figure 9 compare the RFRE as a function of the fault amplitude. From the analysis of the figures, it can be observed that all the techniques accurately estimate the amplitude of either positive or negative faults for a large enough fault amplitude; instead, the estimate is not accurate for small amplitude faults. This is not surprising since when the amplitude of the fault has a magnitude of the same order as the estimator modeling error, the relative fault reconstruction is unreliable and inaccurate. For the $E_{{\%}_{i}}\left(A_{i}\right)$ index, it is not possible to identify a clear winning approach. Indeed, the RBE-Pr method provided the best results for the α(k) sensor, the worst for the β(k) sensor, while for the TaS(k) sensor, the performance was comparable.

## 9. FI Performance on Validation Data (Online Analysis)

**Figure 10:**
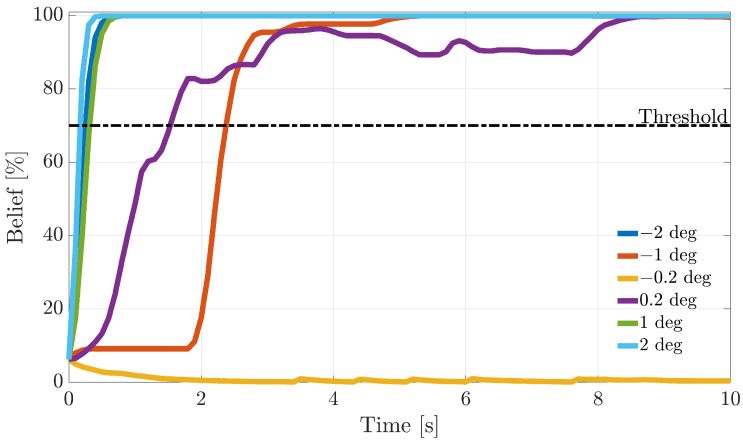
Evolution of the percent fault belief $p[\alpha \left(k\right)|e_{i}\left(k\right)]$ following a failure on the α(k) sensor, for different fault amplitudes (RBE-Pr. method).

**Figure 11:**
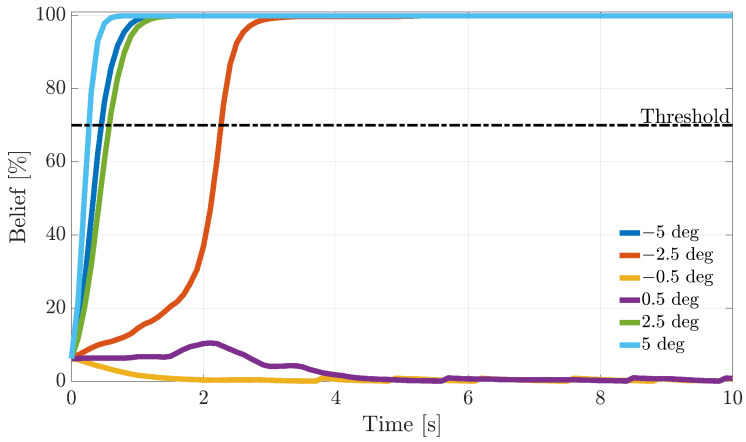
Evolution of the percent fault belief $p[\beta \left(k\right)|e_{i}\left(k\right)]$ following a failure on the β(k) sensor, for different fault amplitudes (RBE-Pr. method).

**Figure 12:**
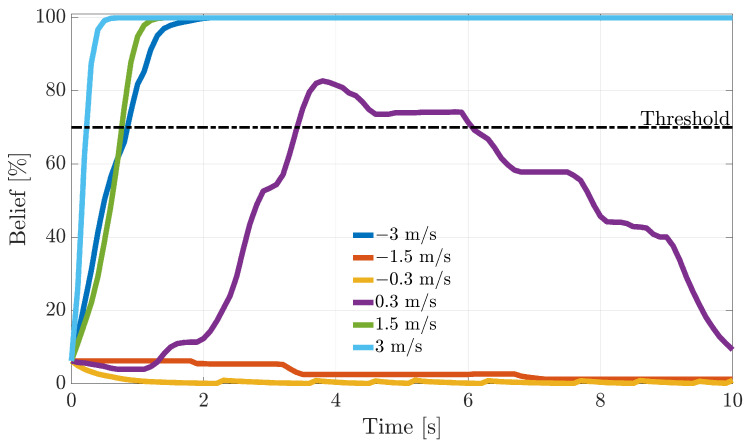
Evolution of the percent fault belief $p[TaS\left(k\right)|e_{i}\left(k\right)]$ following a failure on the TaS(k) sensor, for different fault amplitudes (RBE-Pr. method).

During the flight, following fault detection, it is critical to quickly isolate the faulty sensors and, next, to take the appropriate reconfiguration actions. In this section, the dynamic response of FI methods based on the recursive BFs introduced in Section 5 is analyzed. To understand the online operation in Figure 10, Figure 11 and Figure 12, the evolution of the fault beliefs ($p\left(F_{i}\left(k\right)|e_{i}\left(k\right)\right)$ in (40) for the RBE-Pr technique for a fault injected at $t_{f}$ equal to 50% of the duration of a validation flight is shown. In the figures, the results for fault amplitudes equal to $\pm A_{M}$, $\pm A_{M}/2$, and ± sample-variance of the residuals in fault fault-free conditions are provided.

It is observed that the fault beliefs grow monotonically for large and intermediate fault amplitudes, reaching in a short time the “fault declaration threshold” (set to 70%) when a fault on the *i*-th sensor is declared. On the other side, for a fault amplitude equal to ± the sample variance of the modeling error, the fault beliefs in most cases are not strong enough to reach the threshold; thus, the fault cannot be isolated.

### Fault Isolation Delay

Fault isolation delay quantifies the (mean) time delay between the generic FD and the instant the FI algorithm reaches, for the first time, a fault belief equal to the predefined “fault declaration threshold”. As highlighted in Figure 10, Figure 11 and Figure 12, this delay depends mainly on the fault amplitude, but may depend also on the in-flight fault instant. To evaluate the online FI performance of the schemes, faults (9) were injected into 10 equally-spaced time instants $k_{f}$ for each validation flight. For a fault amplitude $A_{i}$ on the *i*-th sensor, the (mean) fault isolation delay is defined as:

(50) $$T_{isolation_{i}}\left(A_{i}\right)=\sum_{j=1}^{N_{val}}\;\;\sum_{k_{f}\in S_{T_{j}}}^{}T_{isolation_{k_{f}}}\left(A_{i}\right)/\sum_{j=1}^{N_{val}}N_{T_{j}}$$

where:

- $T_{isolation_{k_{f}}}\left(A_{i}\right)$: for a validation flight *j* and for a fault amplitude $A_{i}$, denotes the time from the injection of the fault at $k=k_{f}$ and the time for which the belief associated with the *i*-th sensor reaches for the first time the threshold.
- $S_{T_{j}}$: set of equally-spaced instants ($k=k_{f}$) in the validation flight *j* when the fault (9) is injected in the *i*-th sensor.
- $N_{T_{j}}$: number of samples in the set $S_{T_{j}}$.

**Figure 13:**
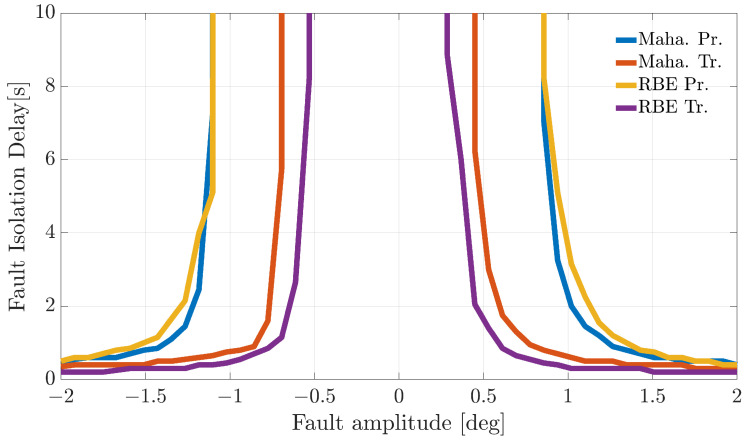
Fault isolation delay for faults on the α(k) evaluated on the validation flights as a function of fault amplitude.

**Figure 14:**
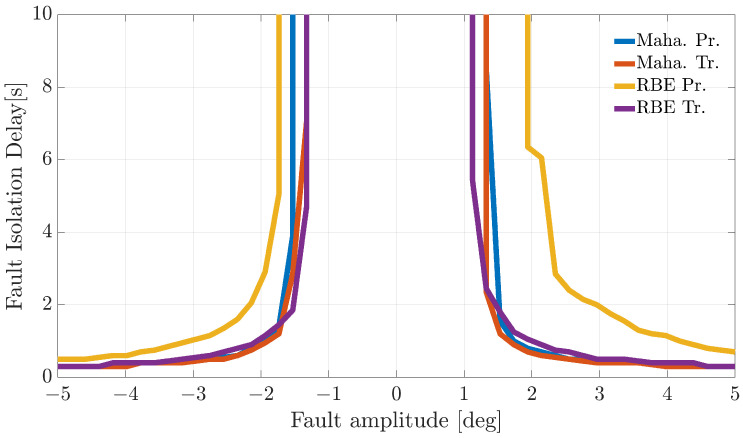
Fault isolation delay for faults on the β(k) evaluated on the validation flights as a function of fault amplitude.

**Figure 15:**
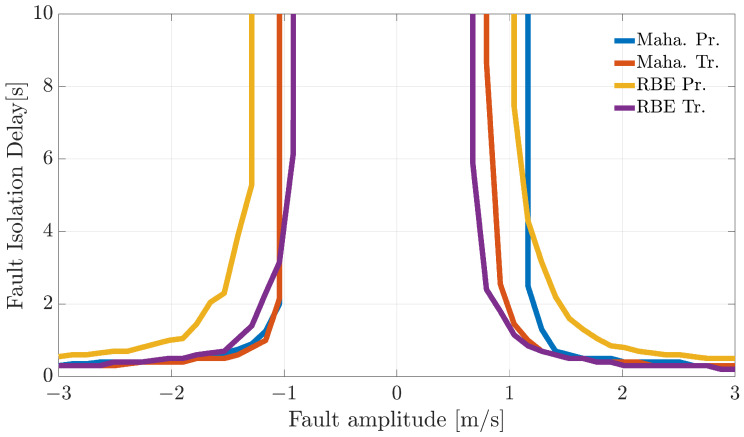
Fault isolation delay for faults on the TaS(k) evaluated on the validation flights as a function of fault amplitude.

Figure 13, Figure 14 and Figure 15 show the index (50) as a function of the fault amplitude. For small-amplitude faults, in case the fault is not isolated within an observation window of 10 s, the corresponding fault isolation time is conventionally set to a very large value. Analyzing the figures, it can be observed that Bayesian filtering for all the methods guarantees a reliable declaration of medium-large faults within 3–4 s. For all three sensors, the best performance (smaller $T_{isolation_{i}}$) was provided by the RBE-Tr. method. Performance degraded quickly for all the methods in the case of small amplitude faults since, in some cases, the fault belief does not reach the fault declaration thresholds within the allowed observation window. Furthermore, in this case, the experimental results confirm that techniques based on transformed directional residuals are more effective (lower mean fault isolation delay) than techniques based on primary directional residuals.

## 10. Conclusions

The purpose of this effort was to compare well-known analytical redundancy-based data-driven techniques for the Fault Isolation (FI) and Fault Estimation (FE) of a set of 14 sensors of a semi-autonomous aircraft. While all these techniques have been shown in the literature to provide close to perfect results using simulated data, only the use of actual experimental data provides the necessary insights and understanding leading to the selection of the best approaches. Specifically, multiple sets of flight data were used to identify linear multivariate models providing a set of primary residuals. Then, residual transformation techniques were applied to generate directional residuals that are robust with respect to the modeling errors. Next, Mahalanobis distance and reconstruction-based methods were used for the FI and the FE. Detailed tests (performed on a set of four validation flights) on the air data sensors showed that the reconstruction-based techniques featuring transformed residuals provide better performance compared to the primary residual-based techniques in terms of the overall fault isolation percentage and fault reconstruction accuracy indices.

In-flight FI was also investigated by applying a bank of recursive Bayesian filters to manage the directional error information online from the 14 sensors. A detailed analysis was conducted by injecting variable amplitude faults at different points throughout the flights. This allowed the estimation of the mean in-flight fault isolation delay. Even for this case, the reconstruction-based methods relying on transformed residuals provided the best performance.

All the considered FI and FE methods are data-driven and were designed based on actual flight data. Therefore, the schemes do not require a priori knowledge of detailed aircraft modeling for their implementation and can be easily returned regularly with updated flight data. Although this is undoubtedly a useful aspect, it is also worth noting, as for any data-based technique, that the reliability of the results depends heavily on the completeness of the available data, which must be representative of all operating conditions. In order to address this issue, all our models were derived by taking multi-flight data rich in maneuvers (as opposed to cruise steady-state conditions), both in the design and validation phases. The quantitative results provide a clear picture of the requested design effort as far as the achievable performance using well-known FI and FE schemes.

## Figures and Tables

**Table 1:** Computational complexity and memory space required by the techniques.

	Maha. Pr.	Maha. Tr.	RBEPr.	RBE Tr.
Computational complexity	$O(n^{3})$	$O(n^{2})$	$O(n^{2})$	$O(n^{2})$
Memory space	$6n_{x}$	$6n_{x}$	$3n_{x}$	$3n_{x}$

**Table 2:** Aircraft sensors.

$x_{0}$				$u_{0}$	
α	Angle of attack	*P*	Roll speed	*Ap*	Aileron position
β	Drifting angle	*Q*	Pitch speed	*Rp*	Rudder position
*TaS*	True air speed	*R*	Yaw speed	*Tp*	Thrust lever position
ϕ	Roll angle	*NNx*	Longitudinal load factor	*Pp*	Pitch trim position
θ	Pitch angle	*NNy*	Lateral load factor	*Sp*	Stabilator position
ψ	Yaw angle	*NNz*	Vertical load factor	*Eng*	Engine revolution
*ZLaser*	Altitude laser	*Zpos*	GPS altitude		

**Table 3:** Selected regressors by the stepwise method for the α, β, and TaS models. TAS, True Air Speed.

Sensor	Selected Regressors (by the Stepwise Method)																
α	β	TaS	NNx	NNy	NNz	P	Q	R	ZLaser	Z	Ap	Rp	Tp	Pp	Sp	Eng	
β	α	TaS	NNx	NNy	NNz	P	R	ϕ	ZLaser	Ap	Tp						
*TaS*	α	β	NNx	NNy	NNz	P	Q	R	ϕ	ψ	Zpos	ZLaser	Ap	Tp	Pp	Sp	Eng

**Table 4:** Number of selected regressors by the stepwise method for the 14 prediction models.

α	β	TAS	NNx	NNy	NNz	P	Q	R	ϕ	θ	ψ	Zpos	ZLaser
16	11	17	16	12	18	14	16	8	9	8	11	15	10

**Table 5:** Maximum fault amplitudes.

	α(k) (deg)	β(k) (deg)	TaS(k) (m/s)
$A_{M}$	2	5	3

## Abbreviations

The following abbreviations are used in this manuscript:

Maha. Pr.	Mahalanobis distance-based technique with Primary residuals
Maha. Tr.	Mahalanobis distance-based technique with Transformed residuals
RBE Pr.	Reconstruction-based technique with Primary residuals
RBE Tr.	Reconstruction-based technique with Transformed residuals
